# Nintedanib in an elderly non-small-cell lung cancer patient with severe steroid-refractory checkpoint inhibitor-related pneumonitis: A case report and literature review

**DOI:** 10.3389/fimmu.2022.1072612

**Published:** 2023-01-10

**Authors:** Lei Pan, Fanqi Meng, Wei Wang, Xu-hao Wang, Hui Shen, Pengchen Bao, Jian Kang, Delei Kong

**Affiliations:** ^1^Department of Respiratory and Critical Care Medicine, The First Hospital of China Medical University, Shenyang, China; ^2^The First Clinical College, China Medical University, Shenyang, China

**Keywords:** checkpoint inhibitor-related pneumonitis, nintedanib, pulmonary fibrosis, non-small-cell lung cancer, immunotherapy

## Abstract

Immune checkpoint inhibitors tremendously improve cancer prognosis; however, severe-grade immune-related adverse events may cause premature death. Current recommendations for checkpoint inhibitor-related pneumonitis (CIP) treatment are mainly about immunosuppressive therapy, and anti-fibrotic agents are also needed, especially for patients with poor response to corticosteroids and a longer pneumonitis course. This is because fibrotic changes play an important role in the pathological evolution of CIP. Here, we report a case demonstrating that nintedanib is a promising candidate drug for CIP management or prevention, as it has potent anti-fibrotic efficacy and a safety profile. Moreover, nintedanib could partially inhibit tumor growth in patients with non-small-cell lung cancer, and its efficacy can be improved in combination with other anti-tumor therapies.

**Highlights:**

- Steroid-refractory CIP in patients with non-small-cell lung cancer (NSCLC) may be particularly lethal owing to progressive respiratory failure and compromised immune system during immunosuppressive treatment of CIP.
- Nintedanib showed great efficacy with good tolerance in treating sustained interstitial fibrosis and reducing pulmonary function decline in the long course of CIP.
- Nintedanib is an anti-tumor-supporting agent in patients with NSCLC, which can positively control tumor growth in combination with chemotherapy and might boost immunotherapy and prevent CIP when used with immune checkpoint inhibitors.

## 1 Introduction

Immune checkpoint inhibitors (ICIs), including programmed cell death 1 (PD-1) or its ligand programmed cell death ligand 1 (PD-L1) and cytotoxic T-lymphocyte antigen 4 (CTLA-4) inhibitors, have made major breakthroughs in improving the progression-free survival (PFS) and overall survival (OS) rates of lung cancer patients (1). The US Food and Drug Administration has moved quickly to incorporate ICIs as a first-line treatment for advanced non-small-cell lung cancer (NSCLC) (2). However, toxicities from ICIs that lead to immune-related adverse events (irAEs) and even fatal adverse events (FAEs) pose a great challenge to immunotherapy (3, 4). Approximately 46.2% of FAEs involves the respiratory system (5, 6). Checkpoint inhibitor-related pneumonitis (CIP) ranges in severity from mild and self-limiting (grade 1–2) to fulminant and life threatening (grade 3–4) and often necessitates immunomodulatory treatments (5, 7, 8). The incidence of any grade CIP in patients with NSCLC ranges from 2% to 39.3% and that for grade ≥3 CIP is approximately 0.6%–4% (9, 10). The mean time from ICI initiation to CIP onset is approximately 10 weeks (2.5 months) (9). The overall fatality rate of any CIP grade ranges from 10% to 27% (5, 10, 11).

Patients with NSCLC are at risk of a higher incidence and severity of CIP (11) owing to increased susceptibility to frequent tobacco exposure (12) and/or underlying chronic respiratory diseases [chronic obstructive pulmonary disease (13), pulmonary fibrosis (14) and tumoral involvement (15)]. Previous real-world studies revealed that patients with NSCLC with CIP had a higher overall response rate but a significantly shorter overall survival after ICI initiation than those without CIP (16–18). Thus, this highlights the importance of better management of CIP to achieve the best outcome of immunotherapy.

Corticosteroids are the core treatment for any grade of CIP according to the current guidelines (19–21). Some patients might be steroid refractory (22) and require other immunosuppressive agents to control the rapid progressive symptoms (especially for those in severe grades) (20), leading to a higher mortality during CIP treatment (12). Some researchers have proposed classifying the clinical phenotype of CIP as acute, subacute, and chronic phases and have divided the pathological process of CIP into inflammatory, profibrotic, and fibrotic stages (23, 24). These concepts provide a new direction for CIP treatment besides immunosuppression therapy, which is anti-fibrotic treatment.

Nintedanib is an oral triple angiokinase inhibitor (25) and has been approved for the anti-fibrotic treatment of patients with idiopathic pulmonary fibrosis (IPF) and chronic interstitial lung diseases (ILDs) (26), even for those with a progressive phenotype (27). Nintedanib is also currently used in patients with coronavirus disease 2019 (COVID-19) to significantly reduce the duration of ventilator use and improve imaging performance (28). In addition, nintedanib has shown sufficient antitumor efficacy in patients with NSCLC (29) and has been approved in the European Union for the treatment of patients with lung adenocarcinoma following first-line chemotherapy (30). Thus, we hypothesized that nintedanib has dual favorable anti-fibrotic and anti-tumor efficacy in patients with NSCLC-CIP and shows good tolerance.

Herein, we report a case of severe steroid-refractory CIP in an elderly patient with NSCLC treated with a PD-1 inhibitor. The patient’s condition was successfully improved by immunosuppression therapy (high-dose corticosteroids and infliximab), medical intensive care unit (MICU) support, and nintedanib administration. We also present a literature review of the contradictory clinical outcomes of CIP in patients with NSCLC and the potential pathogenic mechanisms of CIP. Our findings suggest that nintedanib is a potential anti-fibrotic agent that could act as an important supportive drug in alleviating chronic fibrotic development of CIP and could exert certain antitumor efficacy under critical conditions of severe CIP with good tolerance.

## 2 Case presentation

### 2.1 The basic condition and diagnosis

**Figure 1:**
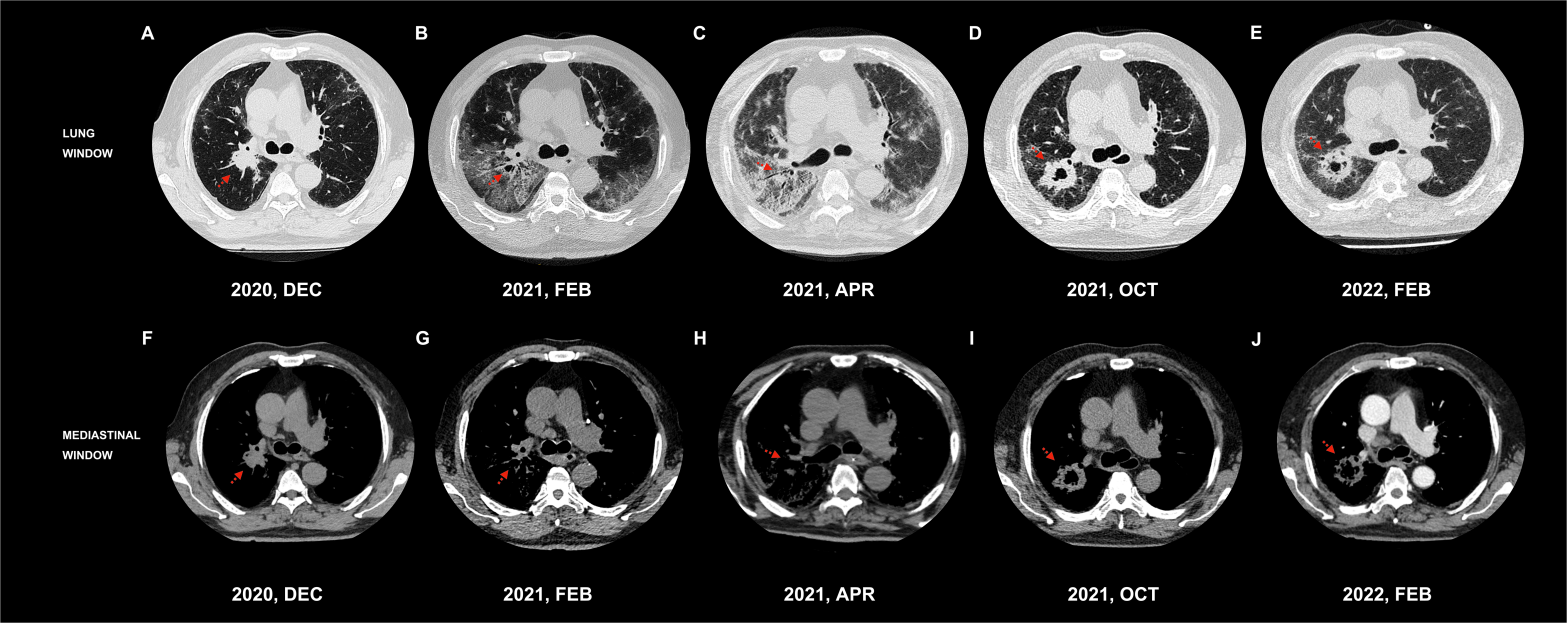
Chest CT of tracheal carina level in the lung and mediastinum windows. dynamic changes of the space-occupying lesions. **(A)**/**(F)** A space-occupying lesion (2.9 × 2.6 cm) in the upper lobe of the right lung. **(B)**/**(G)** The primary foci shrank significantly and was surrounded by GGOs and consolidation shadow. **(C)**/**(H)** Multiple GGOs, reticulation, grid lesions, and traction bronchiectasis in the dominant lobe around the primary foci with patchy lesions in the opposite lobe. **(D)**/**(I)** A recurred lobulated mass (5.0×2.6 cm) in the upper lobe of the right lung. **(E)**/**(J)** Mildly reduced lesion (4.2×4.3 cm).

**Figure 2:**
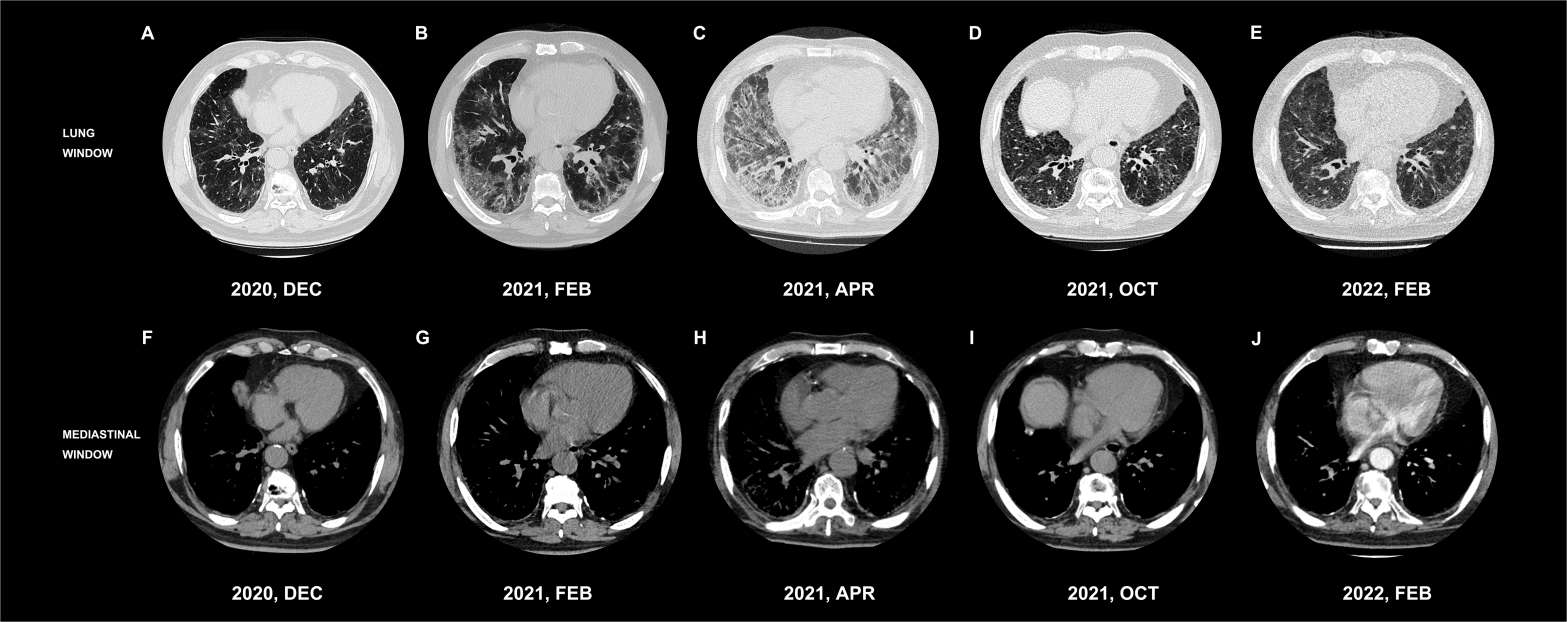
Chest CT of inferior lungs in the lung and mediastinum windows. Dynamic changes in the interstitial lesions in the bilateral inferior lobes of the lung. **(A)**/**(F)** Mild basic interstitial changes. **(B)**/**(G)** Significant peribronchovascular and subpleural GGOs, reticulation, and consolidation. **(C)**/**(H)** Significant fibrotic with GGOs, reticulation, consolidation, honeycomb shadows, and thickened interlobular septa. **(D)**/**(I)** Significantly improved interstitial changes and better transparency. **(E)**/**(J)** Stable mild interstitial changes.

The patient was a 72-year-old male heavy smoker (50 pack-years). During a routine examination in December 2020, a space-occupying lesion (2.9 cm × 2.6 cm) near the lung hilum (**Figures 1A**, **F**) and mild fibrotic changes of unclear origin were detected (**Figures 2A**, **F**). He underwent EBUS-TBNA pathological examination and PET-CT to confirm the final diagnosis of squamous cell lung carcinoma (SqCLC; T1N1MO, stage IIb). Genomic sequencing test showed no driver mutation for targeted therapy (*ALK(−), BRAF(−), BRCA1(−), BRCA2(−), EGFR(−), ERBB2(HER2)(−), FGFR2(−), FGFR3(−), KIT(−), KRAS(−), MET(−), NRAS(−), NTRK1(−), NTRK2 (*–)*, NTRK3(−), PDGFRA(−), RET(−), ROS1(−)*, and *IDH2(+)*); however, the patient tested positive for PD-L1 (TPS=25%, IPS<1%, tested using a Ventana SP263 assay), with tumor mutation burden of 7.26 Muts/Mb (tested using a next-generation sequencing (NGS) panel and paired with peripheral blood sample sequencing). Other genetic mutations related to immunotherapy were also tested as follows: *CD274(−), PDCD1LG2(−), MLH1(−), MSH2(−), MSH6(−), PMS2(−), POLD1(−), POLE(−), TP53(+), ATM(−), ATR(−), BRIP1(−), CHEK2(−), FANCA(−), RAD50(−), PALB2(−), CHEK1(−), MRE11(−), PBRM1(−), MDM2(−), MDM4(−), DNMT3A(−), JAK1(−), JAK2(+), PTEN(−), STK11(−), CCND1(−), FGF19(−), FGF3(−), FGF4(−)*. The patient had no history of cardiopulmonary or connective tissue disease, and the Eastern Cooperative Oncology Group (ECOG) performance status was rated as 2 points.

### 2.2 Two cycles of PD-1 inhibitor + nab-paclitaxel → progressive respiratory failure

**Figure 3:**
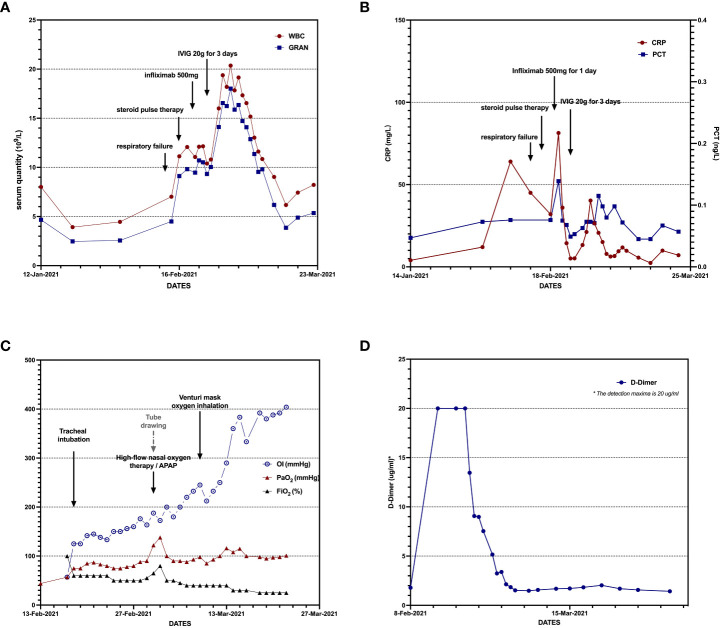
Dynamic changes in laboratory examination: inflammatory reaction, hypoxia with hypercoagulable state **(A–D)**.

Owing to the patient’s age and tumor location, systemic chemotherapy combined with immunotherapy was administered: intravenous infusion of albumin-paclitaxel 160 mg (D1, D8) combined with camrelizumab 200 mg (D1). After two cycles of the above treatment, the patient developed fever after fatigue and colds on 13 February, 2021, with a maximum temperature of 39°C and shortness of breath. The patient was admitted immediately the next day. Bilateral bronchial breathing sounds and Velcro rales were detected. Laboratory examination revealed an inflammatory blood reaction (**Figures 3A, B**), decreased oxygen partial pressure (**Figure 3C**), decreased oxygen saturation (85% SaO_2_), and increased D-dimer levels (≥20 μg/ml) (**Figure 3D**). Images showed that the primary cancer foci shrank significantly and were surrounded by patchy ground-glass opacities (GGOs) and consolidation shadows (**Figures 1B**, **G**). Bilateral peribronchovascular and subpleural GGOs, reticulation, and consolidation were predominant in the middle to lower lungs (**Figures 2B**, **G**). Contrast-filling defects were also observed in the pulmonary artery branches of the upper and lower lobes of the left lung. The patient was preliminarily diagnosed with type I respiratory failure and interstitial pneumonia (CIP?), pulmonary embolism, and SqCLC (T1N1MO stage IIb, partial response [PR]). The patient was administered with mask oxygen inhalation therapy (8 L/min), systemic corticosteroid pulse therapy (methylprednisolone sodium succinate, 240 mg Q.D. for 2 days), and anticoagulation treatment (enoxaparin sodium, 4,000 U Q.D.) on February 16. Considering that infection could not be ruled out, empirical anti-inflammatory and antiviral treatments were administered, along with anti-asthmatic, gastric-protecting, calcium-supplementing, and other symptomatic support treatments. After 48 h, respiratory failure progressively aggravated, and the oxygenation index (PaO2/FiO_2_) was calculated as 125 mmHg. The patient was then transferred to the MICU for further treatment on February 18.

### 2.3 In MICU: Steroid-refractory CIP grade 4 + compromised immune system

**Figure 4:**
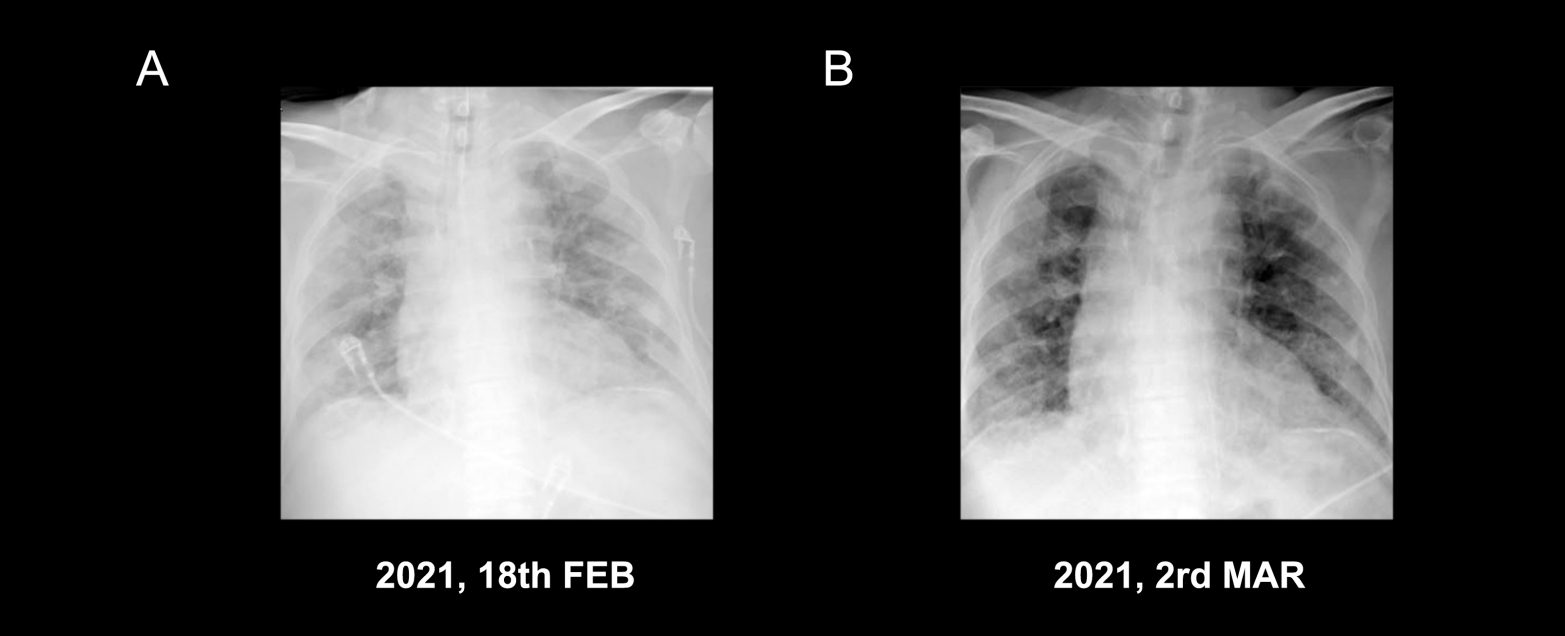
Bedside chest radiograph: both lung translucency and consolidation shadow decreased after treatment **(A, B)**.

The patient was intubated for mechanical ventilation. Bedside chest radiography revealed poor lung transparency (**Figure 4A**). As a diagnosis of exclusion, we considered that the pulmonary symptoms and signs were caused by ICI toxicity and responded inadequately to high-dose corticosteroids. The patient had no signs of other concurrent irAEs, such as dermatitis, hepatitis, nephritis, or myopericarditis. The final diagnosis was steroid-refractory CIP grade 4 (G4), defined as a life-threatening respiratory compromise. For steroid-refractory CIP G4, infliximab (500 mg for 1 day) was administered intravenously, and the ICIs were permanently discontinued. Oral corticosteroids were slowly tempered. Two days after immunomodulatory treatment, the inflammation indicators increased again (**Figures 3A, B**), and plenty of sticky sputum was collected from the patient. We performed sputum culture and sputum smear and found *G+ cocci*, *G− bacillus, Acinetobacter baumannii*, and *Candida albicans.* We then examined the bronchoalveolar lavage fluid (BALF) using NGS and found *Acinetobacter baumannii, C. albicans*, and *Enterocooccusum faecium* infection (**Table 1**).

**Table 1:** Detected bacteria and fungi in BALF by NGS.

Type^a^	Species		Genus	
	Name	Number of detected sequences^b^	Name	Number of detected sequences^b^
G^−^	*Acinetobacter*	221,142	*Acinetobacter baumannii* *Acinetobacter nosocoomialis*	116,450 2,200
G^+^	*Enterococcus*	86	*Enterocooccusum faecium*	33
G^−^	*Dialister*	39	*Dialister pneumosintes* *Dialister invisus*	33 6
G^−^	*Streptobacillus*	27	*Streptobacillus notomytis*	27
G^−^	*Anaeroglobus*	13	*Anaeroglobus geminatus*	13
Fungi	Candida	139	Candida albicans	128

Type ^a^: G^+^ → Gram-positive bacteria/G^−^ → Gram-negative bacteria.

number of detected sequences ^b^: refers to the number of strictly matched sequences of the microorganism detected at the genus/species level.

Considering severe comorbid infectious pneumonia due to compromised immune system, the patient was administered with tigecycline combined with meropenem as anti-inflammatory therapy, micafungin as antifungal therapy, and intravenous immunoglobulin pulse therapy for 3 days to enhance the self-immunological barrier. Enoxaparin sodium anticoagulant therapy was continued for pulmonary embolism. After 3 days, the temperature and laboratory indicators improved (**Figure 3**). Bedside chest radiography showed improved bilateral lung transmittance (**Figure 4B**). On March 2, endotracheal intubation was removed, and the patient was administered with nasal high-flow oxygen (45 L/min, oxygen concentration 50%).

**Figure 5:**
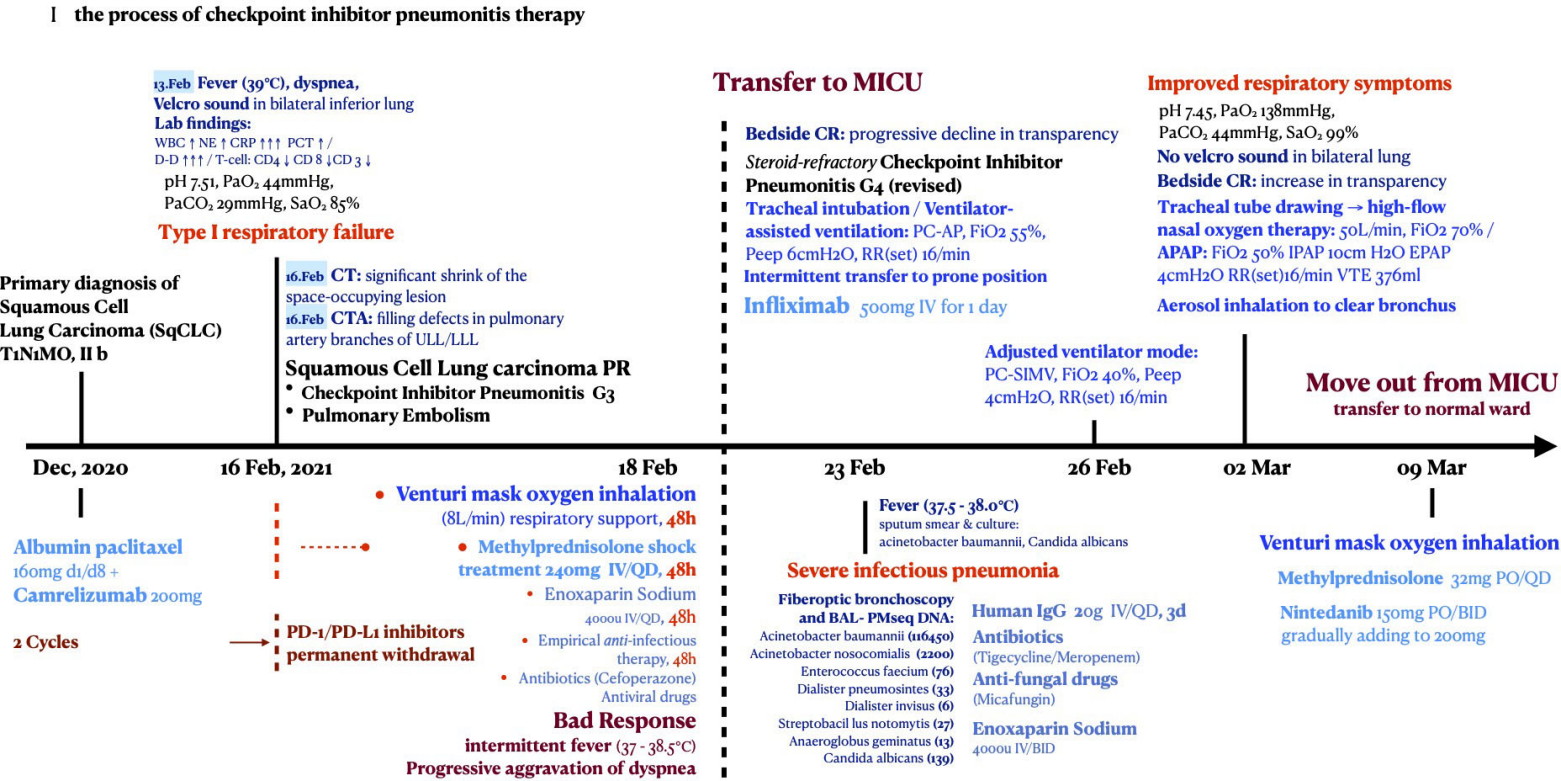
The management axis of acute severe steroid-refractory CIP.

**Figure 5** shows the management axis of CIP G4 and the critical complications in this patient. After sufficient medical care in the MICU, the patient’s condition stabilized, and he was transferred to the general ward on March 9. To manage pulmonary interstitial fibrotic changes and potentially inhibit tumor growth, nintedanib 150 mg was prescribed orally twice daily during the subacute phase of CIP. With good tolerance, the dosage was increased to 200 mg orally twice daily 1 week later and has been maintained to date.

### 2.4 SqCLC maintenance therapy: nab-paclitaxel + nintedanib + afatinib

On 2 April 2021, the patient’s ECOG score was rated 4 before discharge; lung CT reexamination showed no new space-occupying lesions, and the original occupation was significantly smaller than the previous one in December 2020 (**Figures 1C**, **H**). The therapeutic efficacy of SqCLC was evaluated as PR. For CIP, pulmonary injury had evolved into an organizing and fibrotic stage. Multiple GGOs, reticulation, grid lesions, and traction bronchiectasis in a cryptogenic organizing pneumonia (OP) pattern were found in the dominant lobe around the primary tumor site, with patchy lesions in the opposite lobe (**Figures 1C**, **H**). The bilateral lower lungs were predominantly fibrotic with GGOs, reticulation, consolidation, honeycomb shadows, and thickened interlobular septa (**Figures 2C, H**).

Owing to the patient’s deteriorating physical status and COVID-19 public control policy, the patient had recuperated at home for 6 months and maintained oral nintedanib therapy (200 mg twice daily), with slow tapering of oral corticosteroid therapy. The patient visited the hospital for a review on 13 October 2021. The performance status improved, with an ECOG score of 2. The CT scan showed a lobulated mass with a size of 5.0 cm × 2.6 cm in the upper lobe of the right lung, surrounded by fine burrs and thick-walled cavities (**Figures 1D, I**). The interstitial changes in the bilateral lower lobes were significantly improved compared to the previous films (**Figures 2D, I**). There were swollen lymph nodes in the mediastinum, and the largest one was approximately 2.6 cm × 2.0 cm in size. Moreover, the levels of serum tumor markers increased (U/L): CEA, 16.6; AFP, 2.55; CA125, 102; CA153, 36.1; CYFRA, 9.43; NSE, 20.00; and SCCA, 13.6.

The therapeutic efficacy of lung cancer was evaluated as progressive disease (PD), with T2bN2M0 stage IIIa. After consultation with the patient’s family, the third chemotherapeutic cycle, comprising albumin–paclitaxel, was started at 200 mg (D1, D8/q3w), and oral nintedanib treatment was continued. In addition, afatinib 30 mg orally once daily was administered as second-line cancer therapy. The patient received four to seven cycles of albumin-paclitaxel treatment on 11 and 30 November 2021, 22 December 2021, and 21 February 2022.

**Figure 6:**
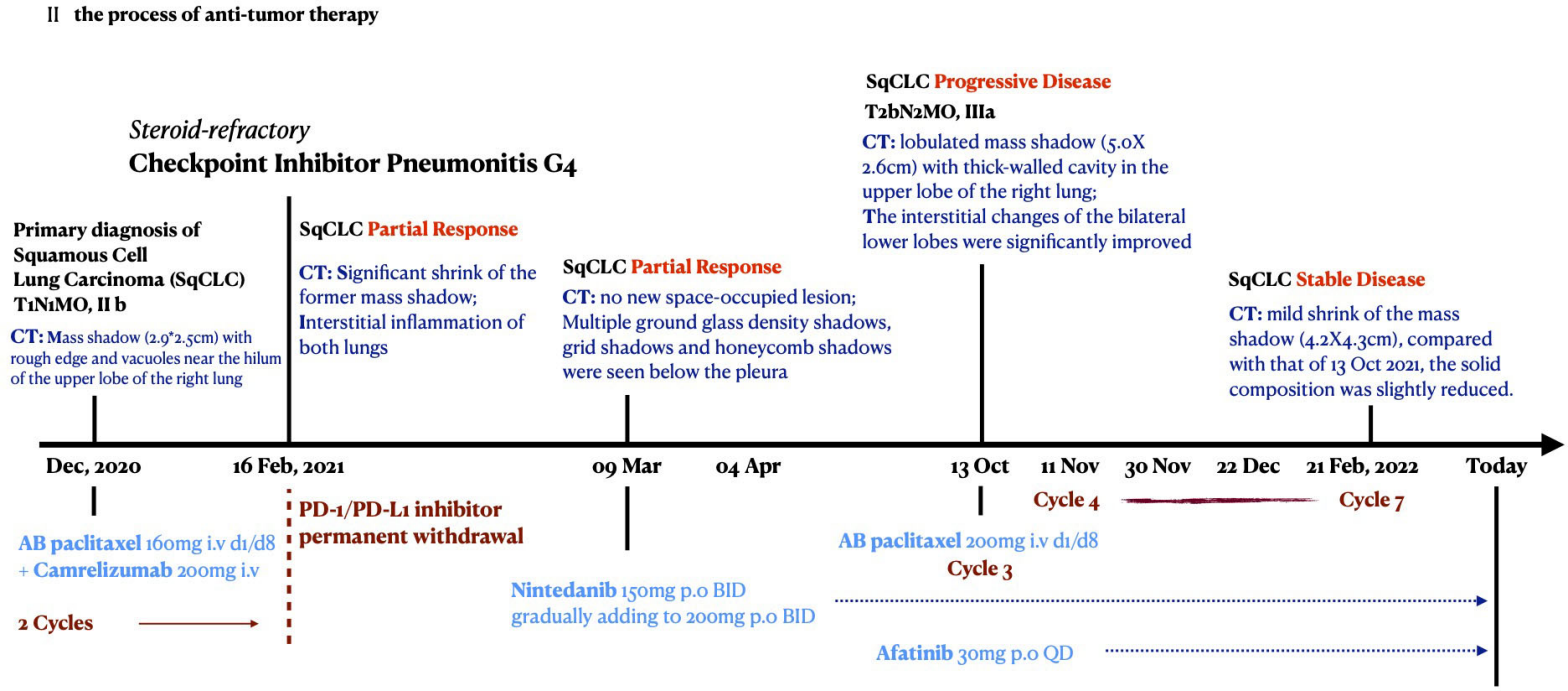
The time axis of anti-tumor therapy.

Lung CT examination showed that the former shadow was 4.2 cm × 4.3 cm in size (**Figures 1E, J**) and stable mild interstitial changes (**Figures 2E, J**). The largest lymph node in the mediastinum was approximately 2.4 cm × 1.8 cm in size. The remaining abdominal CT, brain MRI, and whole-body bone ECT scans showed no obvious abnormalities or metastasis. A response of stable disease was achieved. The patient showed mild adverse reactions: slightly increased levels of liver transaminase (72 U/L AST (15–40) and 69 U/L GGT (10–60). To date, the patient has maintained the current lung cancer treatment regimen. The process of antitumor therapy for the patient is shown in **Figure 6**.

## 3 Discussion

By inhibiting PD-(L)-1 and CTLA-4, ICIs can facilitate immune surveillance and enhance immune attack in the tumor immune microenvironment (12). However, ICI-activated immune mechanisms may attack normal tissues by identifying cross-antigens between normal and tumor cells (31), leading to various types of irAEs (32). The occurrence of irAEs may indicate that immunotherapy has already activated the immune system of patients and is attributed to better tumor clearance effects. A meta-analysis including 4,971 cancer patients found a significant association between irAE occurrence and reduced risk of tumor progression after receiving ICIs (16). Multiple retrospective analyses also reported that the development of irAEs was associated with better survival outcomes in patients with NSCLC treated with PD-(L)-1 inhibitors (17, 33), and patients with greater toxicity to ICIs could attain better tumor-killing effects (18). CIP might occur more often and have a faster onset in NSCLC than in other types of cancer (32). Moreover, CIP is especially associated with better ICI efficacy than any other type of irAE in patients with NSCLC (34). In addition, the occurrence of CIP might suggest that patients with NSCLC can achieve a prolonged tumor-regression duration after ICI discontinuation (18, 35, 36).

However, among the cases of CIP with a variety of malignancies, CIP-related death was mainly observed in patients with NSCLC (15, 37). Two meta-analyses including more than 8,000 patients found that the occurrence of low-grade irAEs/CIP (grade 1–2) was significantly associated with a favorable OS and PFS, whereas severe-grade irAEs/CIP (grade 3–4) was not significantly associated with OS but was significantly associated with a favorable PFS and better overall response rate (16, 33, 36). Low-grade CIP is usually mild and easily manageable, with a shorter pneumonitis course and less impairment of the pulmonary function (11). Severe-grade (with or without steroid-refractory) CIP greatly increases the mortality risk of patients with NSCLC during ICIs treatment owing to overlapping risk factors on the respiratory system (9, 38), compromised pulmonary function (15, 39), and progressive respiratory failure (24), despite the good inhibitory effects of ICIs on the primary tumor.

This contradiction unveils the importance of a better clinical strategy for CIP management (especially severe-grade and/or steroid-refractory CIP) to obtain the best clinical efficacy from immunotherapy in patients with NSCLC. Therefore, a deeper understanding of the onset and progression of CIP and its various clinical manifestations is necessary to identify new treatment strategies. Here, we simply analyzed the pathological mechanisms in different stages of CIP disease progression and the histological and imaging manifestations of each stage. We focused on the mechanisms of the (pro)fibrotic stage in the pathological process of CIP and its possible interacting pathways with the tumor in the lung microenvironment.

Current guidelines for CIP management mainly focus on immunomodulatory therapy to relieve the onset of symptoms. In this case, we used high-dose corticosteroids and infliximab (a chimeric immunoglobulin G (IgG) monoclonal antibody directed at tumor necrosis factor alpha (TNF-α)) to control the rapid progressive respiratory failure of the patient and supported him immediately with critical care in the MICU. He was found to be infected with *Acinetobacter baumannii*, *Candida albicans*, and *Enterocooccusum faecium* in the lung, probably due to a compromised immune system after sufficient immunosuppression and was in a very critical condition. As experienced respiratory physicians, we supported him with flexible mechanical ventilation and prone position ventilation, with comprehensive systemic anti-infective therapy, immunomodulatory therapy, and life-support treatment. This patient improved after 1 week and showed a prominent organizing pneumonia pattern in images with GGOs, consolidation, reticulation, thickened stripes, and grids. However, there is no authoritative definition or treatment recommendation for this chronic fibrotic condition of CIP, especially for steroid-refractory patients, for whom slowly tapering oral corticosteroids cannot play a leading role in further treatment.

Based on our knowledge of the treatment of lung fibrotic disorders and lung cancers, we chose nintedanib as an important supportive drug to manage the fibrotic changes and we hoped that it could potentially inhibit tumor recurrence during recuperation. Herein, we also illustrated previous positive clinical and experimental evidence to support nintedanib as a new therapeutic consideration in CIP management, which has been approved as one of the only anti-fibrotic drugs for progressive interstitial lung diseases (ILDs) (27, 40) and idiopathic pulmonary fibrosis (IPF) (41, 42) and has been recognized as a promising anti-tumor drug for advanced patients with NSCLC (mainly adenocarcinoma) in combination with docetaxel (43, 44). Nintedanib also showed interesting performance in current studies, as it significantly enhanced immune recognition of pulmonary cancerous cells (45, 46) and significantly reduced lung consolidation when combined with immunotherapy (45).

Studies have reported that the incidence of CIP is more often associated with smoking history (47), age over 70 years (38), squamous cell carcinoma subtype(> adenocarcinoma subtype) (31, 48, 49), combination therapy (> ICI monotherapy) (50, 51), PD-1 inhibitors (> PD-L1 and CTLA-4 inhibitors) (51, 52), the presence of baseline fibrosis on chest CT scans (11, 53), and pre-existing pulmonary diseases including IPF, ILDs, chronic obstructive pulmonary disease (COPD), and asthma (14, 13, 54). As we could see, the patient in this case had been exposed to a high risk of CIP and then developed severe steroid-refractory CIP, while we could not provide any preventive treatment before CIP onset, as there were no such recommendations in the previous studies. Nintedanib might be a strong candidate to prevent CIP by applying it in combination with ICIs in patients with NSCLC with a high risk of developing CIP.

### 3.1 Pulmonary injury evolution stages of CIP

The most common symptoms of CIP are non-specific, such as cough, expectoration, and shortness of breath (9, 55). Radiographic features that indirectly reflect the most prominent pulmonary histopathological changes are important for the diagnosis and grading of severity in clinical settings. Despite the huge heterogeneity, some scholars have recognized the following imaging patterns in patients with CIP: acute interstitial pneumonia (AIP), acute respiratory distress syndrome (ARDS), diffuse alveolar damage (DAD), non-fibrotic or fibrotic hypersensitivity pneumonitis (HP), cryptogenic organizing pneumonia (OP), non-specific interstitial pneumonia (NSIP), and other unclassified types (31, 55–57). These patterns can be classified into different evolution phases or different toxicity grades, from the excessive inflammatory stage or the highest grade (AIP/ARDS/DAD) to the proliferative organizing (nonfibrotic HP/OP) and fibrotic stages (fibrotic HP/NSIP) in lower grades (31, 57, 58). In general, all histopathological changes develop from abnormal inflammation resulting from an ICI-activated immune system. Similar to wound healing in lung injuries, mild or severe pulmonary fibrotic changes would always accompany the exaggeration or regression of inflammation (59, 60), which means that inflammatory, organizing, and fibrotic changes could occur simultaneously within the lung tissue (61, 62). The severity and duration of inflammation and the rate of absorption of the damaging exudation determine the clinical and pathological features of CIP in the initial episode before treatment.

The current treatment strategy for CIP mainly involves negative immunomodulatory therapy based on the symptom grade from G1 to G4, including pulse and/or tapering corticosteroids, ICI discontinuation, and immunosuppressant addition (19, 20). Immunosuppressants are usually temporarily applied in steroid-refractory patients who present inadequate response after 48–72 h of corticosteroid treatment (21). Immunosuppressants should be cautiously used, considering their potent immunosuppressive efficacy and unclear negative effects on patients with NSCLC-CIP. First, excessive suppression of the immune system could lead to opportunistic infections very quickly (63–65), which would prolong the duration of critical conditions and increase the complexity of treatment. Second, durable antitumor activity might exist after discontinuation of ICI therapy (35, 66, 67), whereas immunosuppressants, such as infliximab, could weaken the ongoing antitumor immune activity initially launched by ICI treatment (22). As mentioned previously, patients with a severe-grade CIP might achieve better tumor remission, although the clinical symptoms of CIP are life threatening. However, this advantage can be counteracted by CIP treatment, leading to an extremely poor prognosis. These findings are consistent with clinical data. Previous real-world studies have reported that more than half of patients with grade 3 CIP died during ongoing corticosteroid treatment (11) or after receiving additional immunosuppressive drugs (4). In addition, infliximab can cause interstitial lung disease (ILD) (68), which increases the risk of interstitial fibrosis.

In this case, the patient developed secondary *A. baumannii*-induced pneumonia beyond severe-grade CIP and experienced severe interstitial fibrotic changes after receiving high-dose corticosteroids and infliximab. There are no clear recommendations for the sequential maintenance strategy of steroid-refractory CIP patients, especially for those with severe grade. Neither the optimal corticosteroid tapering doses/duration nor further treatment plan for clinical complexity after receiving immunosuppressants is uncertain. This may be attributed to the lack of clinical data caused by premature death in these patients or the lack of consensus on the follow-up pathological mechanism and trends of CIP. Here, we support the longitudinal view of pathological development (23) to divide the pulmonary injury evolution process of CIP into three phases, from inflammation to fibrosis. This separate definition of the fibrotic phase in CIP out of usual pneumonitis grades (G1–G4) typing classification is crucial for treatment: immunosuppressives for inflammation and anti-fibrotics for fibrosis.

#### 3.1.1 Acute inflammatory stage of CIP

Acute exaggerated inflammatory reactions in lung tissues contribute to the progressive reduction in pulmonary function, the primary cause of mortality in patients (69). Histologically, the acute phase of CIP is characterized by acute inflammatory and/or fibrinous exudation in the alveolar cavities (24, 55), accompanied by interstitial inflammatory infiltration and/or fibrosis, which is called mild organization formation (2, 31, 57). Interstitial inflammatory infiltration may include elevated levels of eosinophils, poorly formed granulomas, and lymphocytes (4, 69, 70).

##### 3.1.1.1 Cytotoxic effects of T cells on instigation of CIP

The markedly increased percentage of lymphocytes infiltrating the malignant cells shows the superior tumor immune-clearance effects of ICIs (18). However, enhanced targeted T-cell activity can attack the cross-antigens shared between the tumor and normal lung tissues, leading to off-target toxicity (2, 8, 71). Significant lymphocytosis enriched with CD4+ T cells (72) and CD8+ T cells (70, 73, 74) has been examined in the pulmonary tissues and BAL of patients with CIP, reflecting the participation of lymphocyte-mediated exaggerated immunological reactions. The CD4+ T-cell compartment showed an increase in the number of pathogenic T-helper 17.1 cells, while within the CD8+ T-cell compartment, the number of effector memory T cells were mainly increased (75). An increased proportion of activated autoimmune indicators, CD3+ T cells/HLA-DR + T cells, was associated with the severity grading of CIP (39). Malignant NSCLC tumor cells may have more cross-antigens than normal lung tissues, as they are incubated in the same pulmonary environment (76). Tumor antigens, autoantigens, and neoantigens released from cytotoxic T-lymphocyte-mediated cell lysis induced persistent amplification of immune responses through “epitope spreading” (77). T cells from tumor-infiltrating lymphocytes shared a notable overlap in receptor sequencing with the T cells infiltrating the inflammatory CIP lesions, but not with the secondary lymphoid organs or peripheral blood (78). In addition, decreased expression of PD-1 and CTLA-4 weakened the immune tolerance/anti-inflammatory function of the Treg population (79), whereas the number of central memory T cells with proinflammatory subsets increased (78), which contributes to tumor clearance and the inflammatory microenvironment.

##### 3.1.1.2 Increased proinflammatory cytokines and autoantibodies

Laboratory plasma and BALF examinations of patients with NSCLC-CIP showed an increasing spectrum of common inflammatory cytokines, such as interleukin (IL)-1β, IL-6, IL-17A, IL-35, C-reactive protein, and procalcitonin (72, 76, 80, 81). The levels of surfactant protein-A, surfactant protein-D, and Krebs von den Lungen-6 (KL-6) produced by type II alveolar epithelial cells, which reflect alveolar epithelial cell injury, were also increased (75, 76, 82, 83). These biomarkers usually would decrease to normal levels when the initial respiratory symptoms are relieved. Notably, in addition to the known autoantibodies associated with autoimmune diseases with a higher incidence of CIP, some studies reported a nearly 1.34-fold increase in anti-CD74 plasma level (84) in patients with CIP, while CD74 was related to interstitial pneumonitis (85, 86).

#### 3.1.2 Subacute and chronic phase of CIP: profibrotic and fibrotic stages

For most CIP patients with mild to moderate grades, inflammatory exudation would be absorbed efficiently, with complete weaning off of steroids after 6–8 weeks of initial treatment, leaving mild asymptomatic lesions in the lungs (39, 55). Higher severity and longer duration of inflammation in CIP might exert a great influence on the alveolar microenvironment (72). This could excessively trigger the body’s wound-healing mechanisms (87), leading to persistent reparative attempts by the lung, which manifest as pneumocyte hyperplasia, alveolar epithelial hyperplasia, fibroblastic proliferation, fibrous tissue hyperplasia, alveolar septal thickening, interstitial fibrosis, and lymphocyte infiltration (9, 55, 58, 79). The absorption rate of the lesions is slow or unchanged over an extended period. Even when inflammation is gradually suppressed, activated, and amplified, fibrotic processes may not stop. Chronic CIP was clinically defined as the occurrence of persistent or worsened CIP after steroid taper and CIP recrudescence during steroid taper, which extended the course of immunosuppression to more than 12 weeks (23, 39, 55). During this stage, fibrosing interstitial lung diseases (such as entirely damaged normal lung structure, thickening, and occlusion of blood vessels) are dominant in histology, which might impair air–blood exchange efficiency in the lungs (2, 11, 23). The typical sequelae might be sustained pulmonary interstitial fibrosis and poor pulmonary function caused by severe CIP (39). These patients are more vulnerable to irreversible fibrosis under certain conditions that permanently deteriorate the pulmonary function. Anti-fibrotic agents are thus necessary for this condition.

##### 3.1.2.1 Possible mechanisms in profibrotic and fibrotic stages

Multi-causal fibrotic lung injuries may have pathophysiological mechanisms that overlap with those of idiopathic pulmonary fibrosis, suggesting the potential to share common treatment approaches (59, 88, 89). Early treatment is crucial for slowing the decline in lung function and improving clinical outcomes.

Repetitive injury and reprogramming of the alveolar epithelium (59) are significant triggers of fibrosing progression, inducing an aberrant wound healing response that is characterized by proliferation of fibroblasts and myofibroblasts (61), extracellular matrix (ECM) remodeling (62), and subsequent loss of lung architecture and function (60, 88, 89). Activated fibroblasts and myofibroblasts can secrete ECM components, while myofibroblasts can produce contractile apparatus, such as α-smooth muscle actin microfilaments, which leads to the formation of fibroblastic foci and deposition of ECM (90). Fibroblastic foci in the alveolar parenchyma (91–93) and collagen expansion of the alveolar septa (94) have been detected in CIP lungs. These changes can destroy normal lung structures, causing a characteristic “honeycombing” morphology.

Profibrotic mediators, such as IL-1β (95), transforming growth factor (TGF)-β (96), sphingosine1-phosphate (S1P) (97), and WNT (98) ligands, are involved in this process. TGF-β can induce an unabated form of the fibrotic process called epithelial-to-mesenchymal transition (EMT) (99), in which alveolar epithelial type II cells (undergoing endoplasmic reticulum stress (100), mitochondrial dysfunction (101), or senescence (102) can serve as an unlimited source for the increased myofibroblast-like pool, if the primary inflammatory insult is not attenuated. The microvascular pulmonary endothelium attached to the alveolar epithelium could also become another source of myofibroblasts by endothelial-to-mesenchymal transition (103), which potentially contributes to lung fibrosis and pulmonary hypertension. In addition, immune cells have been detected to infiltrate the chronic inflammatory (91, 93, 94) and fibrotic lesions of CIP. Mast cells (MCs) are innate immune cells that play a key role in the early response to tissue injury (104) and can release an array of profibrotic mediators (105–107), such as histamine, TGF-β (108), MC tryptase (109), and MC chymase (110). MCs would infiltrate immediately the surrounding fibroblast foci, forming a mutually excitatory MC–fibroblast crosstalk in fibrosis that activates the proliferation of MC and fibroblast (106). The number of MCs is positively correlated with the number of fibroblast foci, which has been linked to increased mortality.

##### 3.1.2.2 Crosstalk between pulmonary fibrosis and lung cancer

One particularly special condition in patients with NSCLC-CIP is that both cancer development and immunotherapy adverse effects occur in the lungs. Tumor cells are not isolated but are in constant communication with other cell types within the lung tissue, which forms a particular microenvironment (111). Lung resident fibroblasts surrounding the malignancy are considered first responders to the site of insult that the tumor creates (112), while primary cancerous cells can also infiltrate the abnormal microenvironment that undergoes inflammatory and fibrotic processes of CIP, with the possibility of various cellular active substances and signaling molecules interacting with each other (113, 114).

As early as 1965, the crosstalk between lung cancer and pulmonary fibrosis has been suggested (115), which might pathologically exacerbate their progression (111, 113). The tumor stroma, fibrovascular networks, and tumor microenvironment are vital for the preservation and proliferation of the tumor and its structural integrity. Bronchiolar epithelial cells can undergo reversible EMT (116) during the early stages of carcinogenesis, cancer invasion, and metastasis. Cancer-associated fibroblasts (CAFs) are important players in lung cancer because they exhibit mesenchymal-like features and help build the structure of the tumor stroma (117) with heterogeneous phenotypes. The tumor vasculature can establish fibrovascular networks for the preparation of invasion. Endothelial-to-mesenchymal transition is thought to be the source of 40% of CAFs (118) and may play a role in tumor angiogenic sprouting into adjacent tissues. Through the action of platelet-derived growth factor-BB (PDGF-BB), vascular pericytes released by tumor microvasculature can differentiate into stromal fibroblasts (119), which significantly contributes to tumor invasion and metastasis. Profibrotic mediators, such as TGF-β, are also chronically overexpressed in lung cancer (112). Tyrosine kinase receptor ligands, such as platelet-derived growth factor (PDGF), vascular endothelial growth factor (VEGF), and fibroblast growth factor (FGF), are aberrantly expressed in lung cancer and IPF (114). For example, PDGF plays a critical role in stimulating the secretion of ECM components and growth factors, thereby promoting fibroblast proliferation and recruiting fibrocytes to the lungs.

### 3.2 Nintedanib

Nintedanib (development code: BIBF 1120) is a molecule that does not exist in nature and is a pure synthetic compound synthesized in 1998 during a study on small-molecule inhibitors of angiogenesis (25) [ATC Code: L01XE31 (120)]. Herein, we present the classic concepts and novel experimental results regarding the functional mechanisms of nintedanib to explore its potential efficacy in cancer patients with existing or secondary pulmonary fibrosis (such as CIP).

#### 3.2.1 Mode of action

Receptor tyrosine kinases (RTKs) belong to a group of key enzymes that catalyze important cellular quality and processes, such as cell shape, cell motility, cell cycle control, functional differentiation, gene transcription, and synaptic transmission (121). Abnormal or excessive tyrosine phosphorylation is associated with various cancers (122, 123), inflammatory diseases (124, 125), and pulmonary fibrotic diseases, such as IPF and ILD (126). Therefore, drugs that antagonize related protein tyrosine kinases and phosphatases are expected to control these diseases.

**Figure 7:**
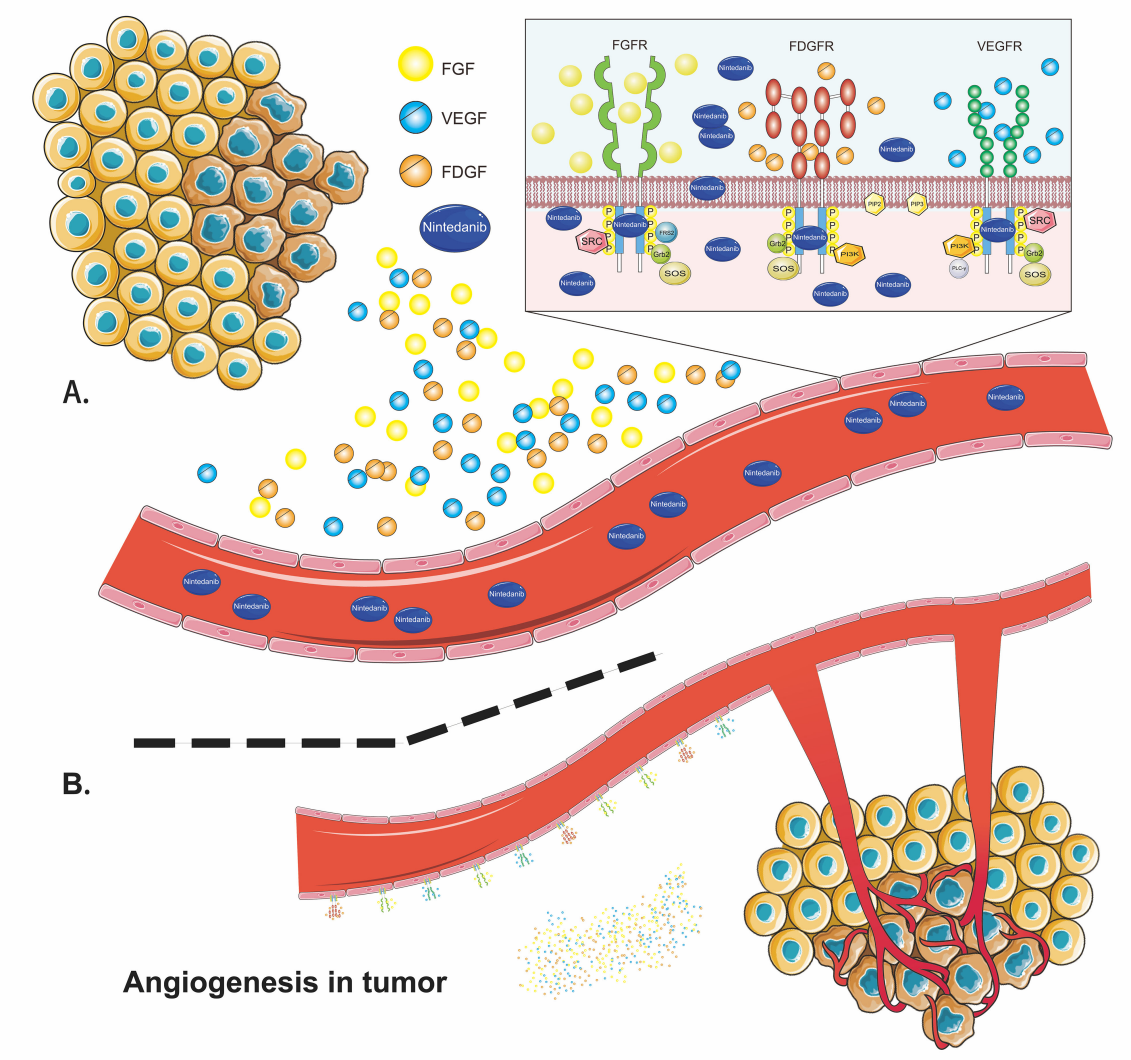
The basic mechanism of nintedanib.

Nintedanib is a small-molecule tyrosine kinase inhibitor that competitively binds to the ATP-binding pocket of kinase to block intracellular signaling cascades (**Figure 7**) (127). It potently inhibits the RTKs implicated in the pathogenesis of various diseases, including vascular endothelial growth factor receptors (VEGFRs), fibroblast growth factor receptors (FGFRs), and platelet-derived growth factor receptors (PDGFRs) (128, 129). Nintedanib also inhibits the receptor tyrosine kinase FLT-3 and non-receptor tyrosine kinases Lck, Lyn, and Src (128), although the contribution of inhibition of these kinases to the therapeutic activity of nintedanib has been less reported (130). In addition to competitively binding to ATP receptor pockets, nintedanib blocks protein kinase activity by allosterically modulating (131) the conformation of ATP-binding sites to inhibit phosphorylation (132). Therefore, nintedanib may exert its anti-angiogenesis, anti-fibrotic, anti-inflammatory, and anti-tumor functions by separately or simultaneously blocking these tyrosine kinases.

The VEGF family binds to VEGFR-1, VEGFR-2, and VEGFR-3 (133). It can promote endothelial cell proliferation and survival, increase vascular permeability, and regulate tumor-related angiogenesis, which is critical for tumor growth and metastasis (134, 135). In NSCLC, elevated VEGF/VEGFR expression is associated with poor prognosis (136). Nintedanib inhibits the proliferation of three types of cells involved in angiogenesis by blocking VEGFRs: endothelial cells, pericytes, and smooth muscle cells (128, 137). There are four types of FGF receptors: FGFR-1, FGFR-2, FGFR-3, and FGFR-4 (138). FGFs play an important role in tissue repair, angiogenesis, inflammation, and tumor initiation and progression (139, 140). Studies have shown that blocking FGFRs can reduce alveolar interstitial fibrosis and inhibit proliferation, migration, and differentiation of fibroblasts to myofibroblasts (141, 142). FGFRs might be anti-tumor therapeutic targets, as FGFR-1 amplification occurs in approximately 20% of patients with SqCLC and is associated with a poor prognosis (143). The PDGF family interacts with homodimers or heterodimers of PDGFR-α/β (144). The PDGF/PDGFR pathway promotes the proliferation, survival, and migration primarily of cells of mesenchymal origin (142). It is associated with fibrosis, cancer proliferation, metastasis, invasion, and angiogenesis (145, 146). Together with FGFRs, PDGFRs regulate the migration and adhesion of pericytes and the transformation of smooth muscle cells to endothelial cells, providing support and stability to vascular walls (144). Nintedanib could reduce the number of blood platelets, block the differentiation of fibroblasts to myofibroblasts, inhibit EMT, and suppress inflammation and angiogenesis by inhibiting these receptors (133, 147).

Pulmonary interstitial inflammatory infiltration in fibrotic lesions may include elevated levels of eosinophils, poorly formed granulomas, and lymphocytes (4). Nintedanib significantly reduced the infiltration of immune cells (including mast cells [MCs] and eosinophils) and neutrophils (147), but not that of macrophages, inhibited granuloma formation (148), and decreased the levels of other proinflammatory cytokines, including IL-4, IL-5, IL-6, and IL-13 (149), during lung fibrosis. Notably, the significant reduction in lymphocyte count by nintedanib was dependent on the early initiation of treatment (150). As mentioned in *Section 3.1.2*, the number of MCs was increased in the region immediately surrounding the fibroblast foci, activating crosstalk with fibroblasts in fibrosis (106). Nintedanib could inhibit recombinant stem cell factor-induced MC survival by directly blocking the phosphorylation of stem cell factor-stimulated c-kit (a type of tyrosine kinase receptor also called stem cell factor receptor) and could reduce the infiltration of MCs (147). Nintedanib also significantly reduced the secretion of TIMP-1, IL-1β, and TGF-β, factors that play key roles in the proinflammatory and profibrotic pathways of pulmonary fibrotic changes (40). Nintedanib reduced pulmonary fibrosis by inhibiting growth-factor-induced proliferation (151) and motility (150) of lung fibroblasts, TGF-β-induced transformation of fibroblasts to myofibroblasts, EMT with increased E-cadherin levels (152), and ECM collagen secretion and ECM deposition (40), and reducing fibrotic gene expression (including collagen 1a1 and fibronectin) (153). Repetitive injury and reprogramming of the lung epithelium are considered critical drivers of fibrosis progression. Nintedanib increased the expression of the transcription factor Nkx2.1 in isolated ATII cells and stabilized the expression of distal lung epithelial cell markers, especially SP-C, to restore normal alveolar epithelial cell function that contributes to its anti-fibrotic effects (153).

Only a few studies have explored the antitumor mechanism of nintedanib. Nintedanib notably increased the ratio of α-smooth muscle actin+/CD31+, a sign of tumor vessel normalization, and reduced distorted vessel density in tumors (45). It also suppressed tumor proliferation by inhibiting CAFs (46, 154) and may inhibit tumor metastasis by blocking EMT (147). Moreover, recent evidence suggests that nintedanib can boost the efficacy of immunotherapy by upregulating MHC-I and PD-L1 expression and increasing the infiltration of CD8^+^ T cells and DC cell maturation to enhance tumor sensitivity to immunotherapy in both *in vivo* and *in vitro* experiments (45, 46), which warrants further clinical evaluation.

#### 3.2.2 Therapeutic efficacy and safety

As a triple angiokinase inhibitor, nintedanib has been clinically studied for pulmonary fibrosis and solid tumors. It is one of the only two drugs currently available to treat IPF (155) and is the only drug approved for use in patients with other progressive fibrosing ILDs and SSc-ILD (156). Nintedanib, in combination with docetaxel, has also been approved by the European Union (30) for the treatment of patients with advanced adenocarcinoma NSCLC. Herein, we summarize previous clinical and experimental evidence of the anti-fibrotic and anti-tumor therapeutic efficacy of nintedanib.

##### 3.2.2.1 In pulmonary fibrosis: potent anti-fibrotic efficacy

Anti-fibrotic drugs, although not curative, can slow ILD progression and have facilitated a paradigm shift in the treatment of IPF over the last decade (150). Nintedanib has been shown to have anti-fibrotic and anti-inflammatory effects in animal models (148) and has shown potent anti-fibrotic effects in well-designed clinical trials of IPF and ILDs (27). It significantly reduced forced vital capacity decline and prolonged the time to first acute exacerbation in patients with IPF (157), irrespective of existing physiological impairment, persistent positive anti-fibrotic effects to slow down IPF progression over more than 4 years of treatment in the INPULSIS trial (42), and improved OS and PFS in the EMPIRE registry (158). Nintedanib has achieved similar effects in reducing annual rates of forced vital capacity decline in patients with chronic fibrosing pulmonary interstitial diseases with a progressive phenotype (such as chronic hypersensitivity pneumonitis and autoimmune ILDs) in the INBUILD (40, 159) and SENSCIS trials (26). Nintedanib has also been shown to reduce lung function deterioration in patients with other fibrotic patterns to reduce lung function deterioration (28, 27, 160).

##### 3.2.2.2 In cancer: Positive anti-tumor efficacy

Nintedanib has shown positive anti-tumor effects in clinical trials on esophageal (161), colon (162), breast (163), prostate (164), and lung cancers, thus attracting increased attention from researchers. Modest disease stabilization was observed in patients undergoing metastatic esophagogastric treatment with nintedanib (161). Nintedanib modulates tumor blood flow and permeability in patients with advanced refractory colorectal cancer (162). In combination with standard chemotherapy, a full dose of nintedanib achieved a 50% pathological complete response in early HER-2-negative breast cancer (163). Nintedanib in combination with afatinib (BIBW 2992), an ErbB family blocker, has shown limited antitumor activity in patients with advanced castration-resistant prostate cancer (164). Combined nintedanib plus bevacizumab treatment achieved a disease control rate of 72.2% in patients with solid tumors (lung, colon, and cervical), even in those with bevacizumab resistance (165).

According to clinical trials of patients with lung cancer, nintedanib monotherapy exhibited relatively inferior efficacy compared to combination therapy (**Table 2**). In 2011, Reck et al. studied the efficacy of nintedanib monotherapy for patients with advanced NSCLC (172). They reported an mPFS of 6.9 weeks, mOS of 21.9 weeks, and disease control rate (DCR) of 49.3% (172). Nintedanib monotherapy has also shown limited activity in relapsed SCLC (173). In later trials, the combination of nintedanib plus pemetrexed achieved a better DCR ranging from 60.9% to 66.7% in patients with advanced recurrent NSCLC (44, 166, 167). Doebele et al. tested nintedanib, in combination with paclitaxel plus carboplatin, in chemotherapy-naive patients with advanced NSCLC and observed a DCR of 84.6% (168). For patients with advanced SqCLC, the LUME-Lung 3 study reported a DCR of 81.3% under first-line combination therapy of nintedanib plus cisplatin plus gemcitabine, with an mPFS of 4.2 months and an mOS of 6.7 months (169). In lung adenocarcinoma, combined nintedanib and docetaxel therapy has been approved as first-line chemotherapy in the European Union (30). The DCR in trials of this combination after first-line chemotherapy ranged from 54.7% to 72.7%, with the longest mPFS and mOS of 5.7 and 12.6 months, respectively (43, 170, 171, 174–176). Three non-interventional studies reported the efficacy of nintedanib and docetaxel after chemo-immunotherapy with the DCR of 78.2%–86% (29, 177, 178). Nintedfanib has shown potent antitumor efficacy combined with chemotherapeutics in patients with NSCLC after first-line chemotherapy or chemo-immunotherapy. As most of these trials were conducted in European countries, more clinical evidence from other regions is warranted.

**Table 2:** From phase I to phase III clinical trials on nintedanib for lung cancers.

Clinical trial (phase)	Reference (details in the annotation)	Year	Patient characteristics	After chemo or chemoimmunotherapy	Patients(n)	Drug combination	Comparator	N dose/frequency	Response n(%)			Disease control	mPFS (mo)	mOS (mo)
									Complete response	Partial response	Stable disease			
I	Ellis et al. (166)	2010	Recurrent NSCLC	previously treated with one first-line platinum-based chemotherapy regimen	26	Pemetrexed + BIBF 1120 (Former Nintedanib)	–	100 or 150 or 200 or 250 mg/bid	1(0.04%)	4(15.4%)	13(50%)	17(65.3%)	5.4	NA
I	Daga et al. (167)	2015	Stage IIIB/IV or recurrent NSCLC	after or failure of prior first-line chemotherapy	18	Pemetrexed + Nintedanib	–	100 or 150 or 200 mg/bid	0	2(11.1%)	10(55.6%)	12(66.7%)	NA	NA
I	Doebele et al. (168)	2012	Chemotherapy-naive advanced NSCLC	as first-line treatment	26	Paclitaxel + carboplatin + Nintedanib	–	50 mg/bid	0	7(26.9%)	15(57.7%)	22(84.6%)	NA	NA
I	LUME-Lung 3 study (169)	2018	Advanced sqNSCLC	as first-line treatment	4+12	Cisplatin + Gemcitabine + Nintedanib (150mg bid)	Cisplatin + Gemcitabine + Nintedanib (200mg bid)	150 or 200 mg/bid	0	5(31.3%)	8(50%)	13(81.3%)	4.2	6.7
I	Okamoto et al. (170)	2015	Stage IIIB/IV or recurrent NSCLC	had received one platinum-based chemotherapy regimen (not containing docetaxel)	38	Docetaxel + Nintedanib	–	100 or 150 or 200 mg/bid	0	10(26.3%)	18(47.3%)	28(73.7%)	5.7	NA
Ib	Yamamoto et al. (171)	2018	Lung adenocarcinoma	after the failure of first-line platinum- based chemotherapy	10	Docetaxel + Nintedanib	–	200 mg/bid	0	4(40%)	3(30%)	7(70%)	NA	NA
II	Reck et al. (172)	2011	Stage IIIB/IV or recurrent NSCLC	after chemotherapy (including one platinum-based chemotherapy)	73	BIBF 1120 (Former Nintedanib)	–	150 or 250 mg/bid	0	1(1.4%)	35(47.9%)	36(49.3%)	6.9w	21.9w
II	Youn Han et al. (173)	2016	relapsed/refractory SCLC	during or after treatment with at least one platinum-based chemotherapy	22	Nintedanib	–	200 mg/bid	0	1(0.05%)	7(31.8%)	8(36%)	1	9.8
II	REFRACT GFPC 02-15 study (174)	2021	Advanced NsqNSCLC	documented progression during first-line chemotherapy based on a platinum-doublet and third- generation drug for ≤ 4 cycles	53	Docetaxel + Nintedanib	–	200 mg/bid	0	10(18.9%)	19(35.8%)	29(54.7%)	2.7	6.9
IIb	SENECA trial (175)	2019	Recurrent NsqNSCLC	received one previous chemotherapy regimen	85+85	Docetaxel (33mg/mq) + Nintedanib	Docetaxel (75mg/mq) + Nintedanib	200 mg/bid	NA	NA	NA	107(63.0%) vs. 124(72.7%)	4.79 vs. 4.82	8.49 vs. 9.62
III	LUME-Lung 1 study (43)	2014	Stage IIIB/IV or recurrent NSCLC	after first-line chemotherapy	655+659	Docetaxel + Nintedanib	Docetaxel + Placebo	200 mg/bid	0 vs. 1 (0.2%)	29(4.4%) vs. 21(3.2%)	325(49.6%) vs. 250(37.9%)	354(54.0%) vs. 272(41.3%)	3.4 vs. 2.7	10.1 vs. 9.1
III	LUME-Lung 1 study (43)	2014	Stage IIIB/IV or recurrent lung adenocarcinoma	after first-line chemotherapy	332+336	Docetaxel + Nintedanib	Docetaxel + Placebo	200 mg/bid	0 vs. 0	15(4.7%) vs. 12(3.6%)	179(55.6%) vs. 136(40.5%)	194(60.2%) vs. 148(44.0%)	4.2 vs. 1.5	12.6 vs. 10.3
III	LUME-Lung 2 study (44)	2016	Stage IIIB/IV or recurrent NsqNSCLC	had received one prior chemotherapy	353+360	Pemetrexed + Nintedanib	Pemetrexed + Placebo	200 mg/bid	0	32(9.1%) vs. 30(8.3%)	183(51.8%) vs. 162(45.0%)	215(60.9%) vs. 192(53.3%)	4.4 vs. 3.6	12.0 vs. 12.7
III	LUME-Lung 2 study (44)	2016	Stage IIIB/IV or recurrent lung adenocarcinoma	had received one prior chemotherapy	335+335	Pemetrexed + Nintedanib	Pemetrexed + Placebo	200 mg/bid	0	32(9.1%) vs. 30(8.3%)	175(52.2%) vs. 153(45.7%)	207(61.8%) vs. 183(54.6%)	4.5 vs. 3.9	12.3 vs. 13.1
III	Cid JR et al. (176)	2016	Advanced lung adenocarcinoma	NA	99	Docetaxel + Nintedanib	–	200 mg/bid	0	27(27.2%)	52(52.5%)	79(79.6%)	NA	NA
*Non-interventional study	Corral et al. (29)	2019	Advanced lung adenocarcinoma after chemotherapy and immunotherapy	After chemo-immunotherapy	11	Docetaxel + Nintedanib	–	200mg/bid	0	4(36%)	5(46%)	9(82%)	3.2	NA
*Non-interventional study	VARGADO (177)	2022	Advanced lung adenocarcinoma after chemotherapy and immunotherapy	After chemo-immunotherapy	80	Docetaxel + Nintedanib	–	200mg/bid	1(1.6%)	31(48.4%)	23(35.9%)	55(86%)(total=64)	6.4	12.1
*Non-interventional study	LUME-BioNIS study (178)	2020	Advanced lung adenocarcinoma after chemotherapy and immunotherapy	After chemo-immunotherapy	55	Docetaxel + Nintedanib	–	200mg/bid	0	10(18.2%)	33(60%)	43(78.2%)	4.6	8.8

##### 3.2.2.3 In patients with NSCLC-CIP: Potential of dual anti-fibrotic and anti-tumor efficacy

Whereas baseline pulmonary fibrosis diseases, such as IPF and ILD, are considered to limit ICI application and severely impair the survival outcomes of patients with NSCLC, nintedanib therapy is a valuable strategy for patients with cancer, complicated with preexisting pulmonary fibrosis. Fukunaga et al. reported that nintedanib inhibited the growth of a newly discovered nodule for 9 months during AE-IPF treatment; the tumor started to increase in size 4 months after the cessation of nintedanib, and the patient was diagnosed with squamous cell carcinoma (41). Nintedanib can exert dual anti-fibrotic and anti-tumor effects in patients with NSCLC with baseline pulmonary fibrosis. Shiratori et al. recently reported that nintedanib monotherapy successfully relieved pulmonary fibrosis and achieved remission of the primary tumor and pleural disseminations in an elderly patient with NSCLC complicated by IPF when he could not tolerate cytotoxic chemotherapy due to IPF progression (179).

According to immunotherapy, a significantly higher incidence rate (10) of any grade of CIP has been reported in patients with NSCLC with preexisting ILD. Unspecified baseline fibrosis was the strongest independent predictor of CIP in patients with NSCLC (11), compared with pertinent demographics and PD-L1 expression. Risk factors for chronic pulmonary fibrosis diseases (180) considerably overlap with those for lung cancer, which may account for the increased burden of CIP in lung cancer patients with preexisting pulmonary fibrosis. However, some of these overlapping risk markers, such as smoking history, are associated with a better clinical outcome in patients with NSCLC treated with ICIs (181); this may explain the favorable clinical overall response rate of immunotherapy observed in patients with NSCLC with preexisting ILD (182–186), as most had a history of smoking. Another meta-analysis that included 10 East Asian studies pooled a significantly higher overall response rate and DCR after PD-(L)-1 inhibitor treatment in patients with preexisting ILD than in those without ILD (10). The reported favorable efficacy might sway the common belief that preexisting pulmonary fibrosis is a contraindication for immunotherapy. It might be an appropriate choice to combine nintedanib with immunotherapy for patients with preexisting pulmonary fibrosis.

In addition, the occurrence of CIP many be strongly correlated with a better tumor remission outcome, as mentioned previously at the beginning of *Section 3*. Beyond baseline fibrosis, patients with NSCLC with a smoking history, older age, squamous cell carcinoma subtype, and pre-existing pulmonary diseases, and those using PD-1 inhibitor were more likely to be at risk of CIP. Thus, patients with a higher risk of developing CIP, especially those with preexisting pulmonary fibrosis, should be closely monitored during ICI therapy or after ICI termination and should be given early diagnosis and intervention under a multidisciplinary approach to obtain the best clinical efficacy from ICIs. Notably, CIP may develop months after ICIs discontinuation (187). Nintedanib could slow down the decline rate of forced vital capacity (40) and potentially strengthen the prevention of CIP (188) when used in combination with ICIs or after ICIs in cancer patients.

Beyond its potential to alleviate pulmonary fibrosis and overcome ICI side effects, nintedanib can increase the efficacy of immunotherapy and overcome ICI resistance (46, 45). The synergistic effect of nintedanib and anti-PD-1 therapy on lung cancers was revealed in *in vitro* and *in vivo* experiments (45); significantly increased ICI therapy responses, relieved aggravated lung injuries, inhibited tumor metastasis, and activated tumor immune microenvironment were observed (46). Nintedanib upregulated the expression of PD-L1 and MHC-I in tumor cells, boosted the immunological recognition and immune clearance of tumor cells, and increased the infiltration and activation of immune cells in tumor tissues, thus improving the efficacy of immunotherapy and overcoming partial ICI resistance (45). In addition, it significantly reduced pulmonary fibrosis in the nintedanib+anti-PD-L1 group, whereas evident pulmonary consolidation was observed in the control and anti-PD-L1 groups (45). These results suggest the dual potential of nintedanib to boost the tumor clearance effects of immunotherapy and prevent or alleviate the development of CIP when applied in combination with ICIs.

A current monocentric phase Ib cohort that studied the safety and efficacy of nintedanib in combination with pembrolizumab in patients with advanced solid tumors reported no case of CIP after combined treatment (189), and nintedanib increased Treg recruitment and circulation of helper memory T cells in the tumor microenvironment to enhance the tumoral immune response (189). These findings confirm the observed antitumor efficacy of nintedanib independent of angiokinase activity (130), which warrants further investigations to explore the antitumor usage of nintedanib in combination with immunotherapy.

Pharmacokinetic studies have shown that nintedanib is rapidly absorbed after oral administration. The pharmacokinetics of nintedanib are time independent and dose linear (172). In patients with advanced cancer, twice-daily administration could achieve a stable state for up to 7 days (127, 190), with a terminal half-life of 7–19 h (129). The cumulative effect of repeated administration was negligible (190). However, current studies have demonstrated that nintedanib can act against a wide range of diseases; however, its low bioavailability is a future challenge for maximizing its effects (191).

In clinical trials, nintedanib has a controlled safety profile, in combination with docetaxel (192), pemetrexed (166), paclitaxel/carboplatin (168), and afatinib (193), with a maximum tolerated dose of 200 mg B.I.D. Blockade of VEGF leads to decreased platelet activity and decreased leukocyte adhesion (194). It has anticoagulant effects and increases the risk of bleeding and thrombosis (165). In addition, blocking PDGF-α and PDGF-β can lead to thrombocytopenia by affecting platelet production (161). Adverse events associated with anti-angiogenic agents in cancer therapy include thromboembolic events, gastrointestinal perforation, bleeding, hypertension, and proteinuria (194). In SqCLCs, bevacizumab (a monoclonal antibody that targets the VEGFR ligand) is not recommended because of the increased risk of severe or even fatal pulmonary hemorrhage (195). Other anti-VEGF small-molecule TKIs, sorafenib and sunitinib, can induce skin-associated adverse events (135), and sorafenib, in combination with carboplatin and paclitaxel, increases the risk of death (196).

Nintedanib is an antiangiogenic agent with an acceptable safety profile and good tolerance in patients with cancer (174). In clinical trials, gastrointestinal reactions, such as mild nausea or vomiting, were the most common adverse events associated with nintedanib (189, 192). Although nintedanib blocks VEGF and PDGF receptors, resulting in a potentially increased risk of bleeding, there is no increased incidence of cardiovascular or bleeding complications observed in large clinical trials involving nintedanib (43, 157, 175). It should be noted that clinical trials have excluded patients with a bleeding tendency during enrollment, which might have resulted in biased results. Moreover, a post-marketing data review showed that <5% of 6,758 patients treated with nintedanib experienced adverse bleeding events, and <1% of patients experienced major bleeding events for approximately 1 year (30). Bleeding events most often involve the digestive, respiratory, and central nervous systems (42). Mild epistaxis was the most common bleeding event (177).

The most common drug-related adverse reactions in patients with advanced NSCLC treated with nintedanib monotherapy were nausea (57.5%), diarrhea (47.9%), vomiting (42.5%), anorexia (28.8%), abdominal pain (13.7%), and reversible elevation of alanine aminotransferase (13.7%) and aspartate aminotransferase (9.6%) levels (172). In the LUME-BioNIS study, the most common on-treatment ADRs/adverse events of nintedanib plus docetaxel were diarrhea (32.3%), malignant neoplasm progression (29.2%), and nausea (15.4%) (178). A phase II study of nintedanib therapy in relapsing small cell lung cancer identified elevated AST/ALT levels and neutropenia as major causes of treatment delay and subsequent dose adjustment (173).

In the present case, the elderly patient showed good tolerance to nintedanib monotherapy (200 mg B.I.D) for long-term treatment and had no adverse hemorrhagic reactions, except for slight dizziness and nausea after nintedanib initiation, which improved later.

#### 3.2.3 Potential of nintedanib in patients with NSCLC with G3-G4 CIP

##### 3.2.3.1 Dual management advantages in critical stage of CIP with good tolerance

Patients who experienced deteriorated or maintained CIP were significantly more likely to have a poor prognosis than those who experienced improved or resolved CIP (9, 11). Those who experienced G3–G4 CIP had to quit ICI therapy to avoid lethal respiratory failure (20), and their physicians tended to hesitate or delay commencement or continuation of aggressive anti-tumor treatment owing to deteriorating physical status, abrasive pulmonary symptoms, and prolonged immunosuppressive management for CIP. Moreover, some immunosuppressive agents, such as infliximab, might weaken the ongoing antitumor immune activity initially launched by ICI treatment (22) during this critical stage.

Nintedanib, with its good safety profile, may be a potent candidate to fill this gap by inhibiting tumor growth under critical conditions and alleviating potential pulmonary fibrosis caused by CIP pathogenesis. However, its efficacy and safety in CIP treatment have only been reported in one case. Xie et al. reported a case of CIP caused by pembrolizumab in a patient with NSCLC (adenocarcinoma) who was successfully treated with nintedanib. However, the patient experienced rapid tumor progression after 2 weeks of nintedanib monotherapy and died 2 months later (83). The patient in this study also showed significant tumor progression after 25 weeks of monotherapy with nintedanib, which must be controlled with other combination therapies.

##### 3.2.3.2 Combination with other anti-tumor therapies in NSCLC

Long-term nintedanib monotherapy was not sufficiently effective to inhibit tumor progression in patients with NSCLC-CIP; however, it significantly ameliorated pulmonary fibrosis, reduced pulmonary function decline, and restored physical activity levels. Thus, physicians should combine other antitumor therapies with nintedanib after physical strength recovery. Here, we chose afatinib as second-line therapy, in conjunction with albumin–paclitaxel chemotherapy, leading to stabilized tumor progression with good tolerance of this case.

Afatinib is a broad-spectrum irreversible blocker of the ErbB receptor family that inhibits the EGFR (ErbB-1/HER1) signaling pathway, ErbB-2/neu/HER2, ErbB-3/HER3, and ErbB-4/HER4 (197). Afatinib has a broader inhibition spectrum than first-generation reversible EGFR-specific drugs, such as erlotinib and gefitinib (198). Although mutations in EGFR/ErbB-1/HER1 have rarely been identified, this pathway has been reported to play a role in the pathophysiology of SqCLC (199). Afatinib can be used as first-line treatment for patients with EGFR mutation-positive NSCLC and has shown considerable efficacy (200). However, given the scarcity of EGFR mutations (<4%) in SqCLCs (201, 202), most patients with advanced SqCLCs do not receive EGFR-TKIs, resulting in paucity of relevant clinical data. Compared with those of first-generation EGFR-TKIs (erlotinib/gefitinib), preclinical data of afatinib showed a lower IC50 value, greater potency capacity against wild-type EGFR antibodies, and the ability to inhibit ErbB-2/neu/HER2 (203–205).

EGFR/ErbB-1/HER1 overexpression occurs in >50% of NSCLCs, especially in >80% of SqCLC tissues (206, 207). The gene copy number of EGFR increases in >25% of patients with SqCLC (199). In addition to EGFR, other ErbB-2/neu/HER2 and ErbB-3/HER3 proteins are overexpressed in more than 20% of SqCLC patients (208, 209). These indicate the potential efficacy of afatinib in mutation-negative patients with SqCLC. The LUX-Lung 8 study reported that the efficacy of afatinib in squamous NSCLC is independent of EGFR mutations (210). Two other phase II studies also showed that afatinib was effective in patients with advanced NSCLC with wild-type EGFR (211).

## 4 Conclusion

Severe-grade (with or without steroid-refractory) CIP is particularly worrisome and is potentially lethal in patients with NSCLC. Sufficient immunomodulator therapy to suppress progressive respiratory symptoms could trigger secondary infectious pneumonia and further deteriorate pulmonary function during CIP treatment. The MICU successfully rescued the case patient from danger. Pulmonary fibrosis, a key stage in the pathological evolution of CIP, warrants the use of anti-fibrotic agents beyond immunosuppressive agents for CIP. Nintedanib, a triple tyrosine kinase inhibitor mostly applied in ILDs, IPF, and NSCLC-adenocarcinoma, has shown potent anti-fibrotic efficacy in the case patient with severe steroid-refractory CIP; however, it has insufficient long-term anti-tumor efficacy by monotherapy and thus requires combination with other anti-tumor therapies to boost efficacy. The dual anti-fibrotic and anti-tumor effects of nintedanib on patients with NSCLC with CIP are worthy of further investigation with clinical trials. It might be a good supportive choice for patients with NSCLC and severe CIP to inhibit tumor and fibrosis progression when they cannot tolerate further toxic anti-tumor therapy.

## Ethics statement

Written informed consent was obtained from the patient for publication of this case report and any accompanying data and images.

## Author contributions

LP and FM: conceptualization, methodology, writing-editing, data curation, and writing—original draft preparation, visualization. WW: supervision and writing—reviewing. X-hW, HS, and PB: visualization, Investigation. JK: supervision, validation. D-lK: supervision and writing—reviewing and editing. All authors contributed to the article and approved the submitted version.

## Glossary

**Table:**

NSCLC	non-small cell lung cancer
CIP	checkpoint inhibitor-related pneumonitis
irAEs	immune-related adverse events
PD-1	programmed cell death-1
PD-L1	programmed cell death ligand-1
CTLA-4	cytotoxic T-lymphocyte antigen 4
PFS	progression-free survival
OS	overall survival
FAEs	fatal adverse events
MICU	medical intensive care unit
IPF	idiopathic pulmonary fibrosis
ILDs	interstitial lung diseases
SqCLC	squamous cell lung carcinoma
NGS	next-generation sequencing
ECOG	Eastern Cooperative Oncology Group
GGOs	ground-glass opacities
PR	partial response
G1–4	grades 1–4
BALF	bronchoalveolar lavage fluid
IVIG	intravenous immunoglobulin
OP	organizing pneumonia
PD	progressive disease
TIME	tumor immune microenvironment
COPD	chronic obstructive pulmonary disease
AIP	acute interstitial pneumonia
ARDS	acute respiratory distress syndrome
DAD	diffuse alveolar damage
HP	hypersensitivity pneumonitis
NSIP	nonspecific interstitial pneumonia
CRP	C-reactive protein
PCT	procalcitonin
SP-A	surface protein-A
ECM	extracellular matrix
α-SMA	α-smooth muscle actin
TGF	transforming growth factor
S1P	sphingosine1-phosphate
EMT	pithelial-to-mesenchymal transition
AT	alveolar epithelial type
EnMT	endothelial-to-mesenchymal transition
MCs	mast cells
CAFs	cancer-associated fibroblasts
PDGF	platelet-derived growth factor
VEGF	vascular endothelial growth factor
FGF	fibroblast growth factor
RTKs	receptor tyrosine kinases
VEGFRs	vascular endothelial growth factor receptors
FGFRs	fibroblast growth factor receptors
PDGFRs	platelet-derived growth factor receptors
SCF	stem cell factor
FVC	forced vital capacity
MTD	maximum tolerated dose
AE	adverse events
